# Supplementary material for: Genetic potential for aerobic respiration and denitrification in globally distributed respiratory endosymbionts
Source: Nat Commun. 2024 Nov 8;15:9682. doi: 10.1038/s41467-024-54047-x (PMC11549363; doi:10.1038/s41467-024-54047-x)
Supplement: Supplementary file 1 — Supplementary Information [file 41467_2024_54047_MOESM1_ESM.pdf]

Supplementary Information to:

Genetic potential for aerobic respiration and denitrification in globally distributed respiratory endosymbionts

Daan R. Speth[1,§,#], Linus M. Zeller[1], Jon S. Graf[1], Will A. Overholt[2], Kirsten Küsel[2,3], Jana Milucka[1#]

Affiliations:
1 Department of Biogeochemistry, Max Planck Institute for Marine Microbiology, Bremen, Germany
2 Aquatic Geomicrobiology, Friedrich Schiller University, Jena, Germany
3 Cluster of Excellence Balance of the Microverse, Friedrich Schiller University, Jena, Germany

§ Present address: Division of Microbial Ecology, Centre for Microbiology and Environmental Systems Science, University of Vienna, Vienna, Austria

# Correspondence: daan.speth@univie.ac.at or jmilucka@mpi-bremen.de

Supplementary materials overview:

<u>This document:</u>

Supplementary Notes

Supplementary Table 1 - Genome features of the 5 Azoamicaceae genomes

Supplementary Figure 1 - Concatenated marker gene phylogeny of Gammaproteobacteria

Supplementary Figure 2 - Phylogenies of electron transport chain complexes

Supplementary Figure 3 - Phylogeny of narG

Supplementary Figure 4 - Phylogeny of nirK

Supplementary Figure 5 - Phylogeny of norB

Supplementary Figure 6 - Phylogeny of nosZ

Supplementary Figure 7 - Phylogeny of Plagiopylea 18S rRNA genes

Supplementary Notes

UBA6186 order

The UBA6186 order is an uncultivated clade sister to the Berkiellales order, within the Gammaproteobacteria class. The clade was first defined in a large-scale assembly and binning effort of publicly available metagenomes that labeled the metagenome assembled genomes recovered as "Uncultivated Bacteria and Archaea (UBA)" [1]. As of GTDB release 207, the UBA6186 clade consists of 17 MAGs, representing 9 species clusters [2]. The existing UBA6186 MAGs were recovered from a diverse range of high latitude environments, namely oil sands tailings pond in Alberta, Canada [3], forest soils in Massachusetts, USA [4], wastewater treatment plants in Germany [5] and Canada [6], the Saanich inlet at Vancouver island [7], several freshwater lakes with oxygen stratification in Europe and North America [8,9] and a saline lake in Antarctica [9]. To the best of our knowledge, no experimental studies or comparative genomics analyses of the UBA6186 clade have been published, and our work defines *Ca.* Azoamicus as the type genus of the order.

Justification for the genus and species designation of the novel cMAGs

The five cMAGs clearly form two distinct lineages, with both cMAGs obtained from OHIO (designated *Candidatus* Azoamicus viridis and *Candidatus* Azoamicus soli) grouping with previously described *Candidatus* Azoamicus ciliaticola. The other two cMAGs, obtained from California and Germany and designated *Candidatus* Azosocius agrarius and *Candidatus* Azosocius aquiferis respectively, form a separate branch (Figure 1, Extended Data Figure 1). When comparing the cMAGs using common sequence similarity metrics (16S rRNA gene identity, average nucleotide identity (ANI), and average amino acid Identity (AAI), Supplemental table S4), it is clear that they represent distinct species. The maximum 16S identity, ANI, and AAI (between *Ca.* A. agrarius and *Ca.* A. aquiferis) are 94.2 %, 82.8 %, and 74.6 % respectively,

all well below proposed species boundaries [10,11] (Supplemental Table S3).

Furthermore, it is clear that *Ca.* A. agrarius and *Ca.* A. aquiferis belong to the same genus, as their 16S rRNA identity, ANI and AAI all fall in the range previously proposed for genera [11,12]. For the three cMAGs we designated Azoamicus, the genus designation is a little more complicated. Their pairwise ANI values are similar to the pairwise ANI values with either Azosocius cMAG, indicating that this metric has saturated. The pairwise AAI between the Azoamicus cMAGs (59.4 % - 60.8 %) is slightly higher than their pairwise identity with the Azosocius cMAGs (53.9 % - 55.2 %), as is their pairwise 16S rRNA identity (89.8 % - 92.3 % versus 86.7 % - 88.7 %), agreeing with the phylogenetic analyses (Supplemental Table S3, Figure 1, Extended Data Figure 1). However, the latter two metrics even fall below proposed cutoffs for genus level.

We choose to propose that each of these two lineages represent a single genus, as it is likely that the cMAGs represent fast evolving genomes and thus that the sequence similarity metrics likely overestimate their evolutionary distance. We also noticed that there is extensive synteny between the cMAGs within a lineage, but not between the cMAGs from two different lineages (Supplemental Table S1). We thus propose that conservation of genome structure should be used in addition to sequence similarity metrics to delineate the genus boundaries of these putative endosymbionts.

<u>Description of gene content</u>

As discussed in the main text, 297 genes in the Azoamicaceae pangenome are conserved between the two lineages. In addition to the genes shared between the two lineages, there are 172 genes unique to either Azoamicus or Azosocius (Figure 2b, supplemental table S1). Of these, 107 genes are present only in Azoamicus genome(s), with the majority (57) unique to the largest *Ca.* A. viridis genome, which contains a full gene complement to synthesize lipids from pyruvate (Supplemental table S1). In addition, *Ca.* A. viridis exclusively encodes a system I type cytochrome c maturation pathway. Other Azoamicus-specific genes of note are protein

trafficking genes encoding the signal recognition particle protein (*ffh*), its receptor (*ftsY*), and several genes in the outer membrane assembly complex (*bamBDE*). However, the gene encoding central subunit *bamA* was present in both lineages, lending support for the retention of an outer membrane in these endosymbionts, as previously proposed [13]. Surprisingly, the genes encoding the molybdenum transporter (*modABC*), required for nitrate reductase, are also unique to the Azoamicus genomes. The 66 genes unique to Azosocius include the pathway for heme biosynthesis from glutamyl-tRNA that is present only in *Ca.* A. aquiferis, although the gene for the final reduction reaction (*hemH*) is missing. In addition, notable genes present only on both Azosocius genomes are a second copy of the ADP:ATP translocase required for ATP delivery to the host, as well as the gene for DNA mismatch repair protein mutH.

Supplementary Tables and Figures

Supplementary Table 1 - Genome features of the 5 Azoamicaceae genomes

Organism name	Genome size	Genes	CDS	rRNA	tRNA	tmRNA	GC content (%)	coding density (%)
Azoamicus ciliaticola	292520	350	311	3	35	1	24.4	91
Azoamicus viridis	373572	418	378	3	36	1	25.9	92
Azoamicus soli	284432	336	299	3	34	0	25.1	92
Azosocius agrarius	352496	384	347	3	33	1	22.4	92
Azosocius aquiferis	353051	388	352	3	32	1	20.5	92

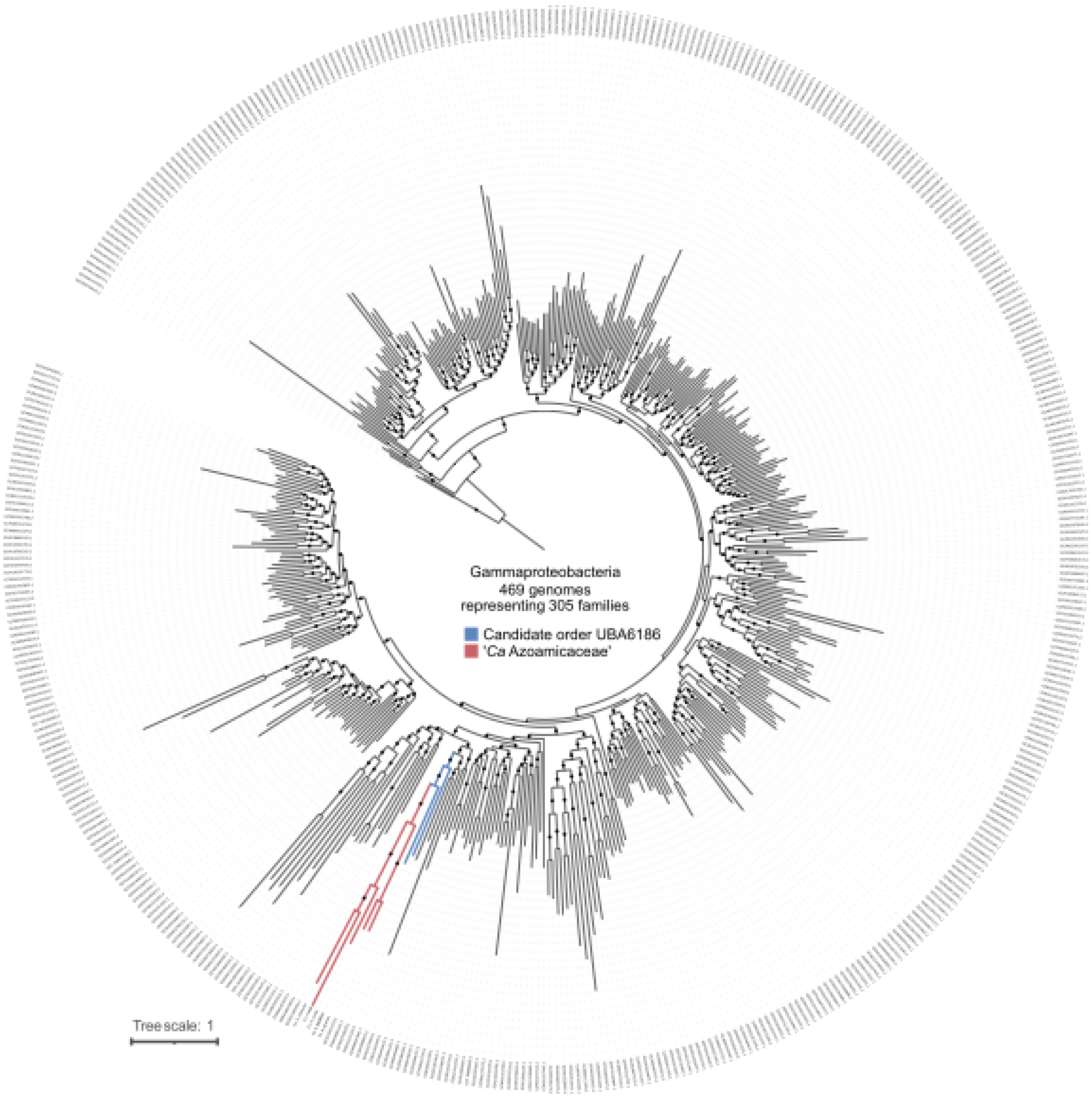

<u>Supplementary Figure 1 - Phylogenetic placement of *Azoamicaceae* within the UBA6186 order in the Gammaproteobacteria class.</u>

Concatenated marker gene phylogeny of representatives of all 305 Gammaproteobacteria families in GTDB r207, including representatives of 2 genera per family if available. 146 families consisted of a single genus and are thus represented by a single genome. Genomes were selected based on type species status and contig number/completeness/redundancy.

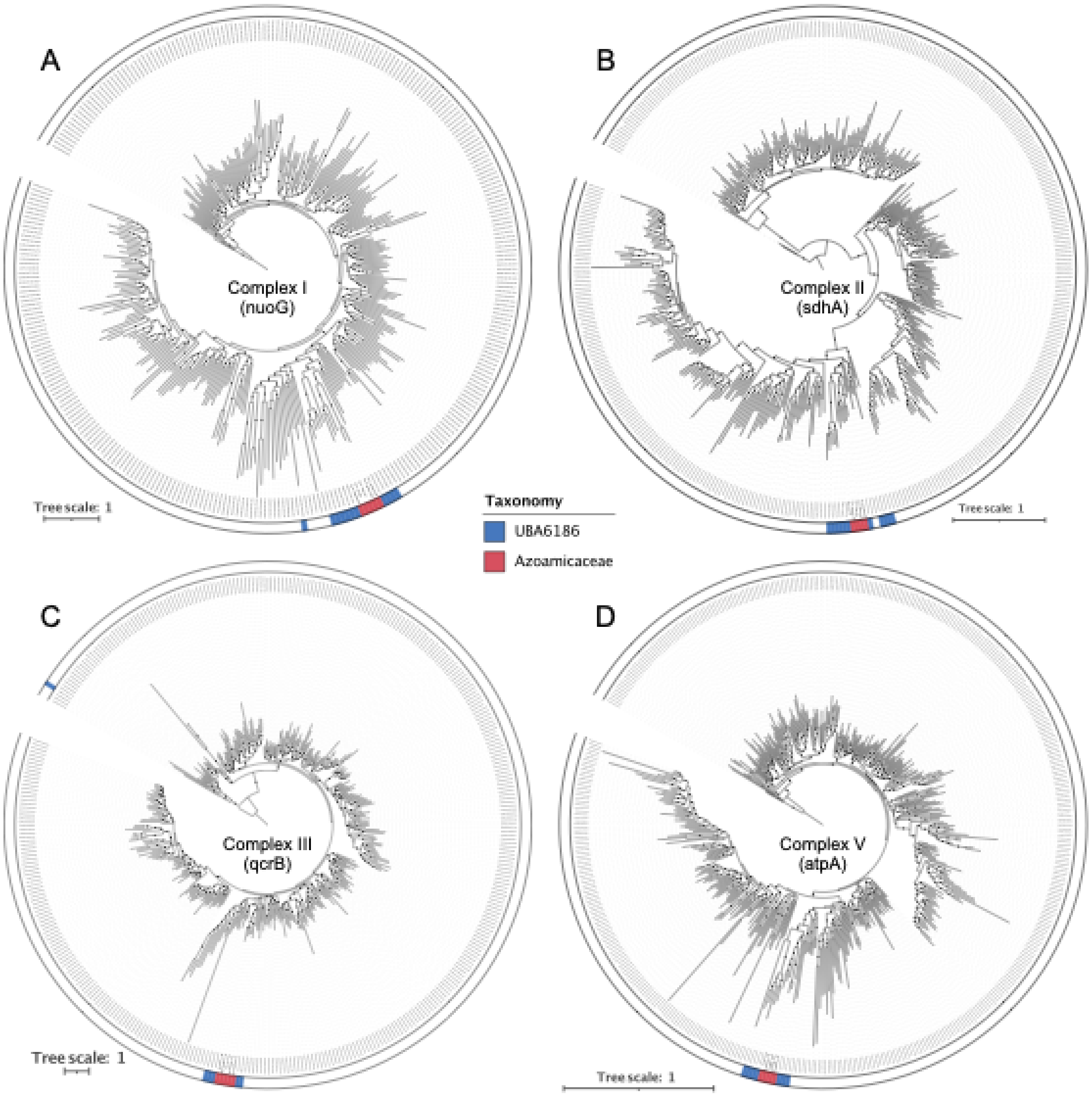

<u>Supplementary Figure 2 - Respiratory chain complexes I, II, III and V are vertically inherited.</u>

Phylogenetic trees of the amino acid sequences of genes encoding subunits of A) complex I (*nuoG*), B) complex II (*sdhA*), C) complex III (*qcrB*), and D) complex V (*atpA*). Reference sequences were obtained from the 305 genomes included in Extended data figure 1, as well as all the UBA6186 genomes included in the genome taxonomy database (GTDB v207; GCA and GCF identifiers) and global catalog of earth's microbiomes (GEM; GEMOTU identifiers) databases. Color strips indicate sequences from the Azoamicaceae family and UBA6186 order. Black circles on the branches indicate bootstrap values higher than 80%.

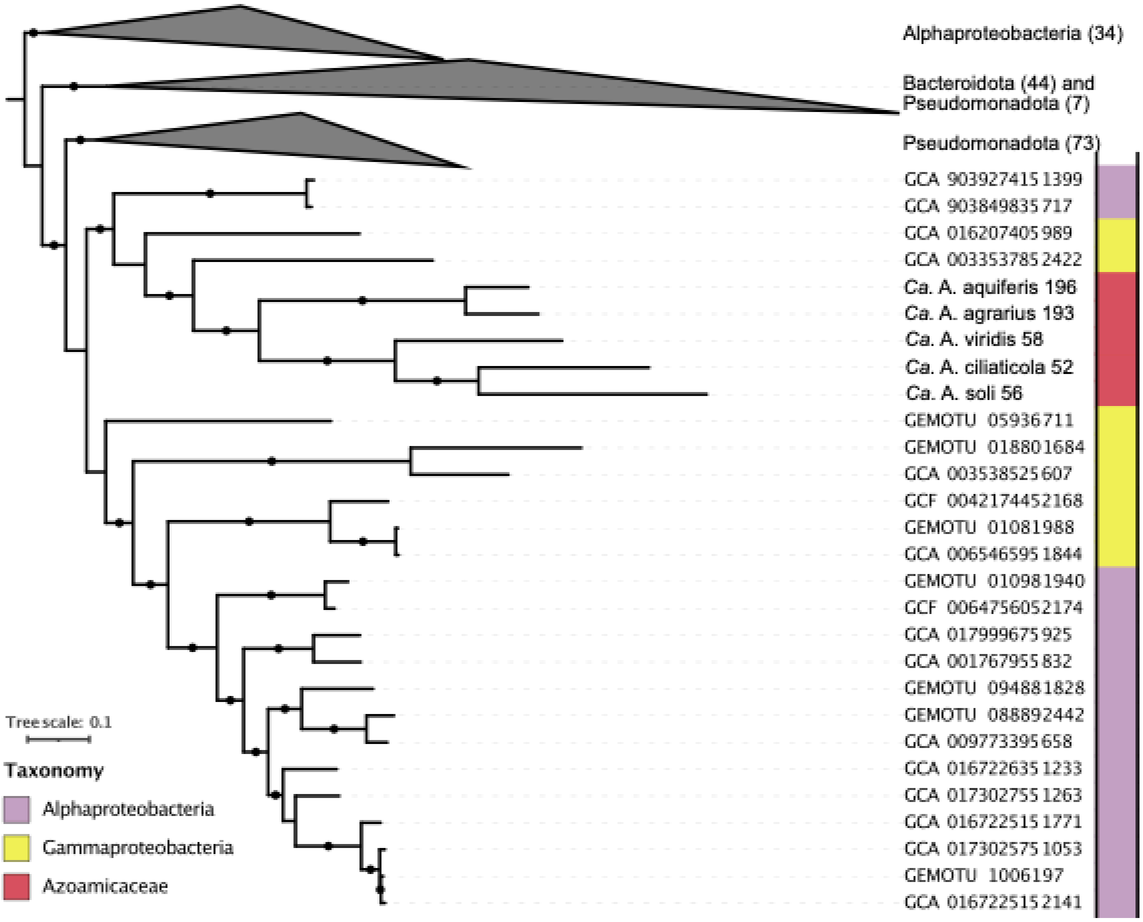

<u>Supplementary Figure 3 - Nitrate reductase phylogeny suggests acquisition via lateral transfer.</u>

Phylogenetic tree of the amino acid sequences of the molybdenum containing subunit of the nitrate reductase (narG). Sequences were obtained from genomes included in the genome taxonomy database (GTDB; GCA and GCF identifiers) and global catalog of earth's microbiomes (GEM; GEMOTU identifiers) databases. Color strip indicates the GTDB assigned phylum (or class for Pseudomonadota) of the genome containing the *narG* gene, with colors consistent between Extended data figures 3-6. Black circles on the branches indicate bootstrap values higher than 80%.

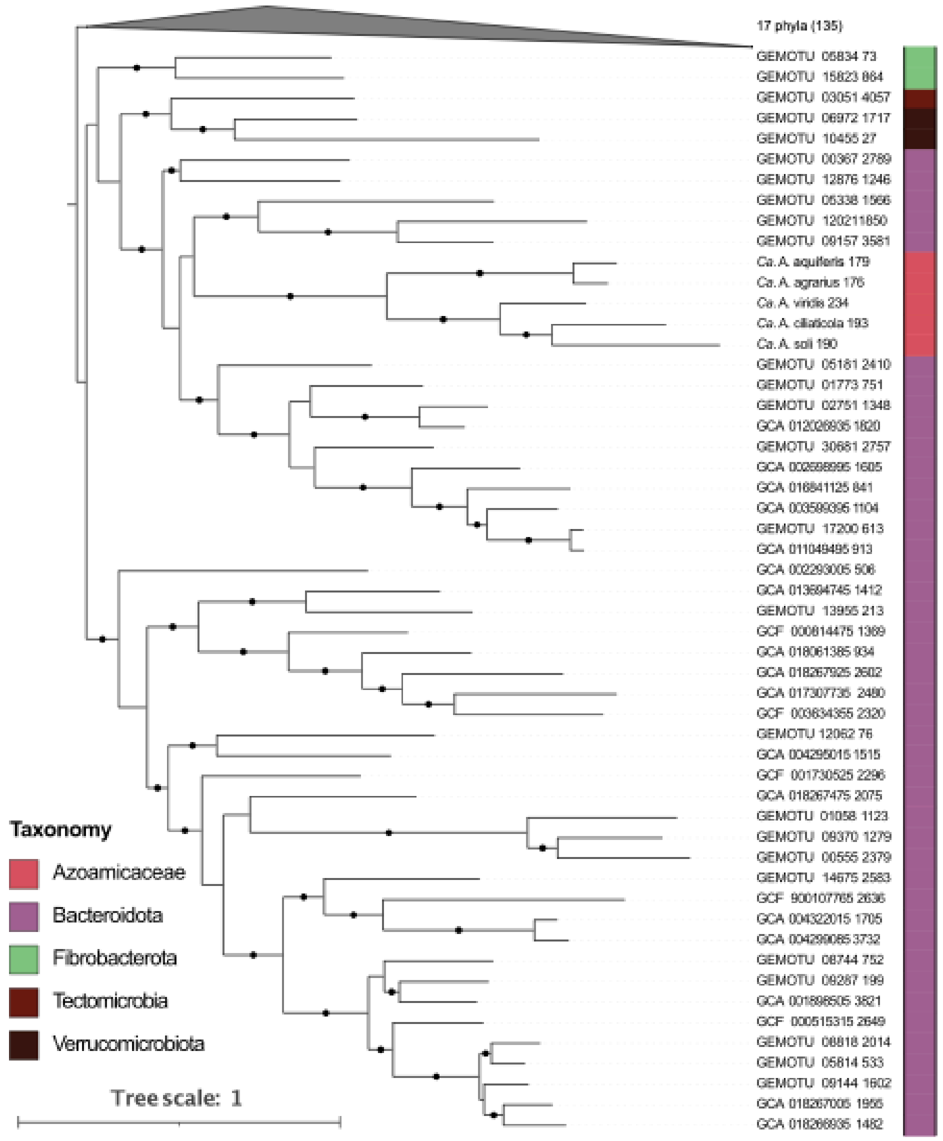

<u>Supplementary Figure 4 - Nitrite reductase phylogeny suggests acquisition from Bacteroidota.</u>

Phylogenetic tree of the amino acid sequences of the copper containing nitrite reductase (nirK). Sequences were obtained from genomes included in the genome taxonomy database (GTDB; GCA and GCF identifiers) and global catalog of earth's microbiomes (GEM; GEMOTU identifiers) databases. Color strip indicates the GTDB assigned phylum (or class for Proteobacteria) of the genome containing the *nirK* gene, with colors consistent between Extended data figures 3-6. Black circles on the branches indicate bootstrap values higher than 80%.

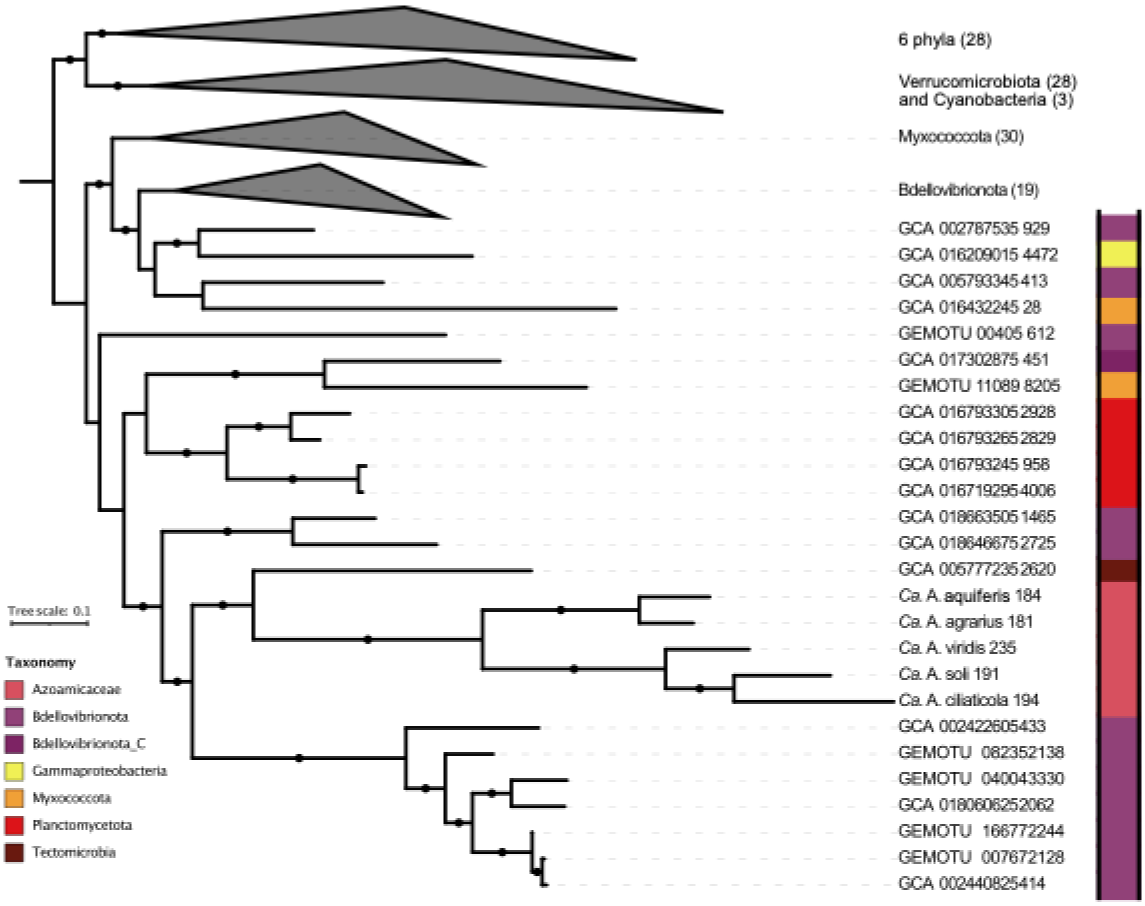

Supplementary Figure 5 - Nitric oxide reductase phylogeny.

Phylogenetic tree of the amino acid sequences of the integral membrane subunit of the C-NOR type nitric oxide reductase (norB). Sequences were obtained from genomes included in the genome taxonomy database (GTDB; GCA and GCF identifiers) and global catalog of earth's microbiomes (GEM; GEMOTU identifiers) databases. Color strip indicates the GTDB assigned phylum (or class for Proteobacteria) of the genome containing the *norB* gene, with colors consistent between Extended data figures 3-6. Black circles on the branches indicate bootstrap values higher than 70%.

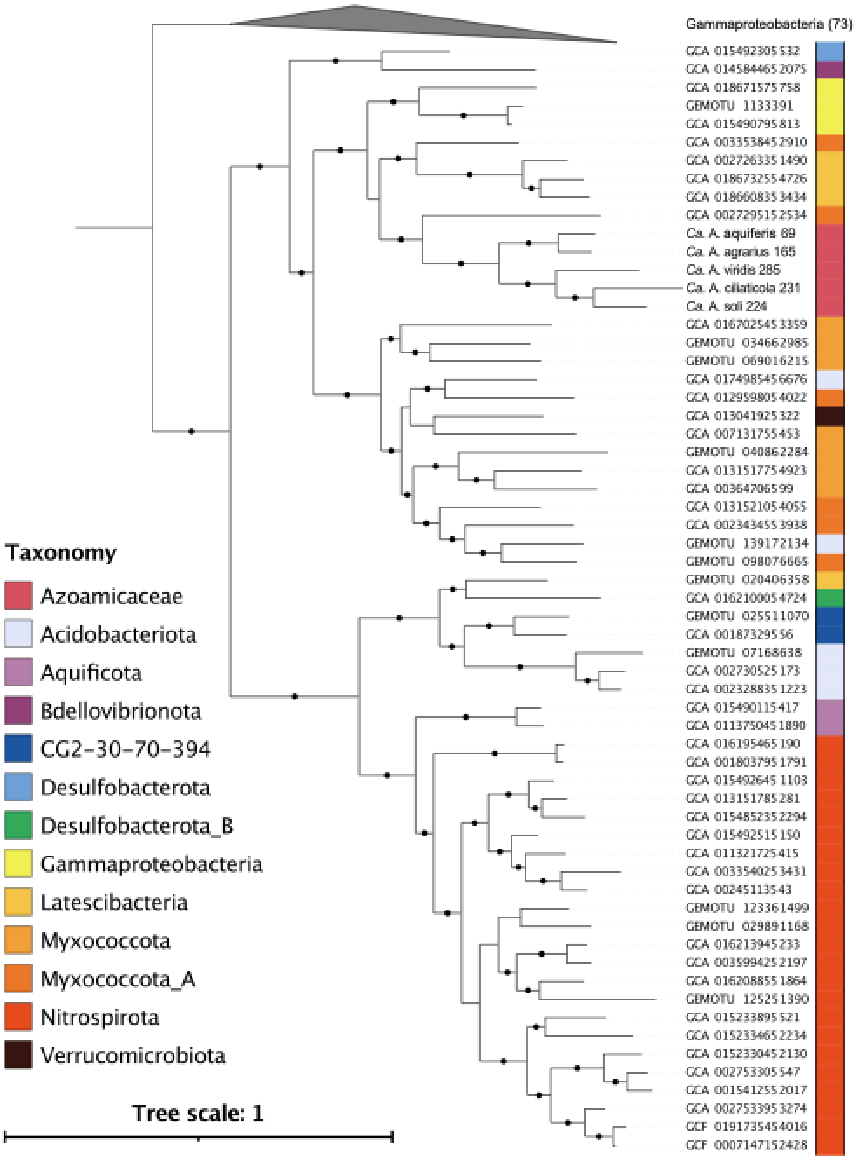

Supplementary Figure 6 - Nitrous oxide reductase phylogeny suggests frequent transfers.

Phylogenetic tree of the amino acid sequences of the catalytic subunit of nitrous oxide reductase (nosZ). Sequences were obtained from genomes included in the genome taxonomy database (GTDB; GCA and GCF identifiers) and global catalog of earth's microbiomes (GEM; GEMOTU identifiers) databases. Color strip indicates the GTDB assigned phylum (or class for Proteobacteria) of the genome containing the *nosZ* gene, with colors consistent between Extended data figures 3-6. Black circles on the branches indicate bootstrap values higher than 80%.

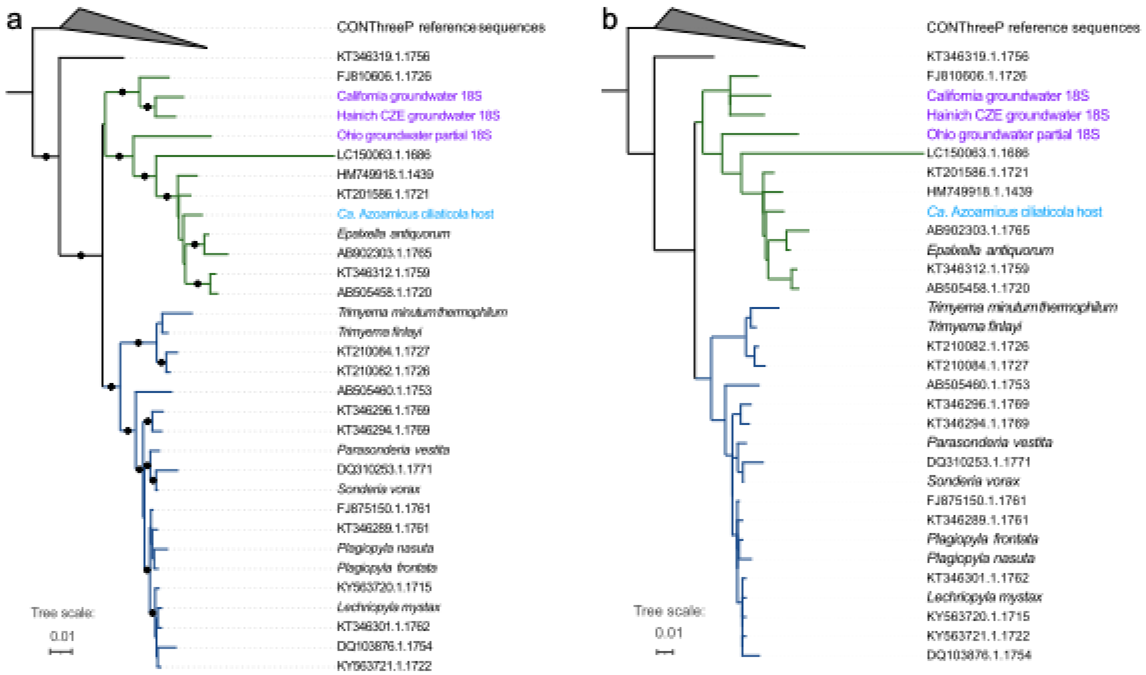

Supplementary Figure 7 - 18S rRNA gene phylogeny of Plagiopylean ciliates.

(a) FastTree2 18S rRNA gene sequence phylogeny of the full length and partial 18S sequences retrieved from the source data for the Azoamicaceae genomes (purple), the confirmed host of Ca. A. ciliaticola (light blue) and reference sequences from the Silva database (version 138.1, black). The expanded section of the tree shows the Plagiopylea class, with the branches of the Plagiopylida order shown in dark blue and the branches of the Odontostomatida order shown in green. Black circles indicate >90% bootstrap support. (b) RAxML placement of the full length and partial 18S sequences retrieved from the source data for the Azoamicaceae genomes (purple) and the confirmed host of Ca. A. ciliaticola (light blue) in a Silva reference phylogeny of the same sequences included in (a).

References

1. Parks, D. H. *et al.* Recovery of nearly 8,000 metagenome-assembled genomes substantially expands the tree of life. *Nat Microbiol* **2**, 1533–1542 (2017).
2. Parks, D. H. *et al.* GTDB: an ongoing census of bacterial and archaeal diversity through a phylogenetically consistent, rank normalized and complete genome-based taxonomy. *Nucleic Acids Res.* **50**, D785–D794 (2022).
3. Tan, B. *et al.* Comparative analysis of metagenomes from three methanogenic hydrocarbon-degrading enrichment cultures with 41 environmental samples. *ISME J.* **9**, 2028–2045 (2015).
4. Alteio, L. V. *et al.* Complementary Metagenomic Approaches Improve Reconstruction of Microbial Diversity in a Forest Soil. *mSystems* **5**, (2020).
5. Schneider, D., Zühlke, D., Poehlein, A., Riedel, K. & Daniel, R. Metagenome-Assembled Genome Sequences from Different Wastewater Treatment Stages in Germany. *Microbiol Resour Announc* **10**, e0050421 (2021).
6. Spasov, E. *et al.* High functional diversity among Nitrospira populations that dominate rotating biological contactor microbial communities in a municipal wastewater treatment plant. *ISME J.* **14**, 1857–1872 (2020).
7. Lin, H. *et al.* Mercury methylation by metabolically versatile and cosmopolitan marine bacteria. *ISME J.* **15**, 1810–1825 (2021).
8. Buck, M. *et al.* Comprehensive dataset of shotgun metagenomes from oxygen stratified freshwater lakes and ponds. *Sci Data* **8**, 131 (2021).
9. Nayfach, S. *et al.* A genomic catalog of Earth's microbiomes. *Nat. Biotechnol.* **39**, 499–509 (2020).
10. Jain, C., Rodriguez-R, L. M., Phillippy, A. M., Konstantinidis, K. T. & Aluru, S. High throughput ANI analysis of 90K prokaryotic genomes reveals clear species boundaries. *Nat.*

Commun. **9**, 5114 (2018).

11. Konstantinidis, K. T. & Tiedje, J. M. Prokaryotic taxonomy and phylogeny in the genomic era: advancements and challenges ahead. *Curr. Opin. Microbiol.* **10**, 504–509 (2007).
12. Barco, R. A. *et al.* A Genus Definition for Bacteria and Archaea Based on a Standard Genome Relatedness Index. *MBio* **11**, (2020).
13. Graf, J. S. *et al.* Anaerobic endosymbiont generates energy for ciliate host by denitrification. *Nature* **591**, 445–450 (2021).
