# Supplementary material for: Genetic potential for aerobic respiration and denitrification in globally distributed respiratory endosymbionts
Source: Nat Commun. 2024 Nov 8;15:9682. doi: 10.1038/s41467-024-54047-x (PMC11549363; doi:10.1038/s41467-024-54047-x)
Supplement: Supplementary file 2 — Description of Additional Supplementary Files [file 41467_2024_54047_MOESM2_ESM.pdf]

File Name: Supplemental_Data_1___Ca_A_agrarius_reoriented.fa.gz
Description: Reformatted contig representing Ca. Azosocius agrarius to start at the predicted origin of replication.

File Name: Supplemental_Data_2___Gene_overview_and_annotation.xlsx
Description: Azoamicaceae cMAG gene annotation and gene presence/absence.

File Name: Supplemental_Data_3___metabolic_predictions.xlsx
Description: Azoamicaceae cMAG metabolic predictions based on KEGG and anvi'o.

File Name: Supplemental_Data_4___similarity_by_16S_ANI_and_AAI.xlsx
Description: Azoamicaceae cMAG average nucleotide identity, average amino acid identity, and 16S rRNA gene similarity.

File Name: Supplemental_Data_5___Azoamicaceae_pseudofinder_prediction.xlsx
Description: Azoamicaceae cMAG predicted pseudogenes using pseudofinder.

File Name: Supplemental_Data_6___Azoamicaceae_SNVs.xlsx
Description: Azoamicaceae cMAG single nucleotide variability as predicted by InStrain.

File Name: Supplemental_Data_7___Hainich_metaranscriptome.xlsx
Description: in situ metatranscriptome analysis of Ca. Azosocius aquiferis.

File Name: Supplemental_Data_8___IMNGS.xlsx
Description: Azoamicaceae IMNGS amplicon analysis results.
